# The Sufferings of Christ

**Published:** 1887-10-29

**Authors:** 


### WORDS OF CONSOLATION.

[Communications for this column should be addressed, "Chaplain," care of the Editor of THE HOSPITAL, who will gratefully welcome any suggestions or inquiries from matrons, nurses, and the staff generally.]

### LVI.—THE SUFFERINGS OF CHRIST.

*(For Reading to the Sick.)*

OUR Blessed Lord's sufferings may be divided into three periods,—(1) the Agony, comprising the time from which He left the supper-table to the arrest by Judas; (2) the period from the arrest until the darkness commenced while He was on the Cross; (3) the last three hours' agony in the darkness. The second of these periods, with which we are now engaged, embraces the worst of His physical sufferings. He suffered in mind through these hours, of course, as well as in body, but no Agony. He was calm and collected. No sign of mental agitation can be traced. He was pained much by St. Peter's denial, by the previous insults heaped upon Him, by false accusations, and the coarsest mockery. Nevertheless we find the details, as related by the evangelists, are chiefly concerned with the pains inflicted upon His body and the members thereof. We have observed, that to render the sacrifice complete suffering seems to have been accepted in every member of the body. It is said that others have suffered as much in this respect, and for longer, than Christ did. Certainly, in some ways, they have. Some of the martyrdoms were very dreadful specimens of the most brutal cruelty possible. We are not sure, though, for the comparatively short time, that we meet, either in history or experience, with any complication of torture like what He had to bear. Now if the body is redeemed from hell as well as the soul, there may be some very strong reasons given why we should expect to meet some such complication of physical suffering in the working out of the Great Atonement. Remember, the greater part of the sufferings to which mankind are heirs through sin, are felt at present in the body. The body is often the first witness to the presence and power of sin. We do not want to be taught in a hospital what fearful complications of suffering are brought about through the lusts of the flesh being wantonly indulged. Sowing unto the flesh means, here at least, reaping corruption. Will this be otherwise hereafter? St. Paul leads us to believe that the corruption, begun in the body through sin, will, if not checked, be perpetuated in hell. "Whatsoever a man soweth *that* (that very same seed) shall he reap" (GAL. vi, 7), unless accepting salvation now, he shall be delivered from the power as well as from the guilt of sin. We shall endeavour to make it plain to you in succeeding articles that the sacrifice of Christ offered in the body provides a remedy for our deliverance from every class of sin done in the body. He not merely made Atonement for sin as a whole, but in the smallest and apparently most trifling details of His Passion He has indicated the lines of self-denial upon which we must tread in order to make the Cross effectual for the rooting out each one of the sins which beset us. Jesus came into the world to save us from our sins—that is, to deliver each one of us, if we will, from the dominion and the effects of our individual misdeeds. Many people seem to think that He came to save them in their sins.

*(To be continued.)*

